# Optimizing responses to drug safety signals in pregnancy: the example of dolutegravir and neural tube defects

**DOI:** 10.1002/jia2.25352

**Published:** 2019-07-12

**Authors:** Lynne M Mofenson, Anton L Pozniak, Jacque Wambui, Elliot Raizes, Andrea Ciaranello, Polly Clayden, Peter Ehrenkranz, Ade Fakoya, Andrew Hill, Saye Khoo, Imelda Mahaka, Surbhi Modi, Cynthia Moore, Andrew Phillips, George Siberry, Kenly Sikwese, Claire Thorne, Heather D Watts, Meg Doherty, Nathan P Ford

**Affiliations:** ^1^ Elizabeth Glaser Pediatric AIDS Foundation Washington DC USA; ^2^ Chelsea and Westminster Hospital NHS Foundation Trust London School of Hygiene and Tropical Medicine London UK; ^3^ National Empowerment Network of people living with HIV/AIDS in Kenya (NEPHAK) Nairobi Kenya; ^4^ Centers for Disease Control and Prevention Atlanta GA USA; ^5^ Massachusetts General Hospital Boston MA USA; ^6^ HIV i‐Base London UK; ^7^ Bill & Melinda Gates Foundation Seattle WA USA; ^8^ Global Fund Geneva Switzerland; ^9^ University of Liverpool Liverpool UK; ^10^ Pangaea Zimbabwe AIDS Trust Harare Zimbabwe; ^11^ University College London London UK; ^12^ US Agency for International Development Arlington VA USA; ^13^ African Community Advisory Board (AFROCAB) Luska Zambia; ^14^ University College London Institute of Child Health London UK; ^15^ Office of the US Global AIDS Coordinator Washington DC USA; ^16^ World Health Organization Geneva Switzerland

**Keywords:** women, ARV, treatment, health systems, neural tube defects, periconception, pharmacovigilance

## Abstract

**Introduction:**

The unexpected identification of a neural tube defect (NTD) safety signal with preconception dolutegravir (DTG) exposure in the Botswana Tsepamo birth outcomes study brought into sharp focus the need for reliable data on use of new antiretrovirals in pregnancy, improved pharmacovigilance systems to evaluate safety of new drugs being introduced into populations including women of reproductive potential, and balanced risk‐benefit messaging when a safety signal is identified.

**Discussion:**

The Tsepamo study NTD safety signal and accompanying regulatory responses led to uncertainty about the most appropriate approach to DTG use among women of reproductive potential, affecting global DTG roll‐out plans, and limiting DTG use in adolescent girls and women. It also revealed a tension between a public health approach to antiretroviral treatment (ART) and individual choice, and highlighted difficulties interpreting and messaging an unexpected safety signal with uncertainty about risk. This difficulty was compounded by the lack of high‐quality data on pregnancy outcomes from women receiving ART outside the Tsepamo surveillance sites and countries other than Botswana, resulting in a prolonged period of uncertainty while data on additional exposures are evaluated to refute or confirm the initial safety signal. We discuss principles for evaluating and introducing new drugs in the general population that would ensure collection of appropriate data to inform drug safety in adolescent girls and women of reproductive potential and minimize confusion about drug use in this population when a safety signal is identified.

**Conclusions:**

The response to a signal suggesting a possible safety risk for a drug used in pregnancy or among women who may become pregnant needs to be rapid and comprehensive. It requires the existence of appropriately designed surveillance systems with broad population coverage; data analyses that examine risk‐benefit trade‐offs in a variety of contexts; guidance to transform this risk‐benefit balance into effective and agreed‐upon policy; involvement of the affected community and other key stakeholders; and a communication plan for all levels of knowledge and complexity. Implementation of this proposed framework for responding to safety signals is needed to ensure that any drug used in pregnancy can be rapidly and appropriately evaluated should a serious safety alert arise.

**Abbreviations**
APR: Antiretroviral Pregnancy Registry
ART: antiretroviral therapy
DTG: Dolutegravir
EFV: efavirenz
EPPICC: European Pregnancy and Pediatric HIV Cohort Collaboration
FAERS: U.S. Food and Drug Administration FDA Adverse Event Reporting System
GDG: WHO Guidelines Development Group
HIV: human immunodeficiency virus
NTD: neural tube defect
PEPFAR: Presidents Emergency Plan for AIDS Relief
WHO: World Health Organization

## 1 Introduction

While there is an urgency to bring newer and more potent antiretroviral drugs developed in resource‐rich settings to resource‐limited settings as quickly as possible, evaluation of the safety of new drugs in pregnancy is often limited to small pharmacokinetic studies following drug approval. Detection of less common adverse events in pregnancy, such as birth defects, requires evaluation of a large number of exposures, which only occurs when the antiretroviral drug is introduced into populations including women of reproductive potential, principally in resource‐limited settings. There is a critical need for reliable data on the safety of new drugs in pregnancy, improved pharmacovigilance systems in resource‐limited settings to evaluate the safety of new drugs that will be widely used by women of reproductive potential, and balanced risk‐benefit messaging when a safety signal is identified.

These needs have been brought into sharp focus by the identification of a neural tube defect (NTD) safety signal with preconception dolutegravir (DTG) exposure in the Botswana Tsepamo birth outcomes study. The Tsepamo study was initially developed to assess whether efavirenz (EFV)‐based antiretroviral therapy (ART) use at the time of conception was associated with increased NTD risk. It was designed to compare pregnancy outcomes in women living with HIV with those in women without HIV infection and, for women living with HIV, to compare pregnancy outcomes by ART regimen and timing (initiated preconception versus during pregnancy) 1. After Botswana modified their national guidelines in 2016 to recommend DTG‐based ART as the preferred first‐line regimen for adults, the study added objectives to evaluate pregnancy outcomes in women receiving DTG. Early data on the safety of DTG initiated during pregnancy was reassuring 2.

In May 2018, the World Health Organization (WHO) Guidelines Development Group (GDG) was planning to recommend transition of most people living with HIV to DTG‐based ART, a regimen with improved efficacy and better tolerability and durability compared to many other ART regimens. It was hoped this change would lead to improved viral suppression rates at the individual, community and global level, resulting in decreased global HIV incidence 3. Because the Tsepamo study was within a few months of evaluating their primary EFV research question, the investigators agreed to perform and share with the WHO GDG an early unplanned analysis of pregnancy outcomes (planned for August 2019) with preconception DTG initiation, to complement their previously reported data on DTG initiated during pregnancy.

Unexpectedly, the preliminary results demonstrated a small but statistically significantly increase in absolute NTD risk in infants born to women receiving DTG‐based ART at the time of conception (4 NTD/426 exposures, prevalence 0.94%, 95% confidence interval (CI) 0.37%, 2.4%) compared to infants born to women receiving non‐DTG‐based ART at the time of conception (prevalence 0.12%, 95% CI 0.07%, 0.21%), receiving EFV‐based ART at time of conception (prevalence 0.05%, 95% CI 0.02%, 0.15%), initiating DTG‐based ART during pregnancy (prevalence 0.00%, 95% CI 0.00%, 0.13%) and women without HIV (prevalence 0.09%, 95% CI 0.07%, 0.12%) 4. The public release of a safety alert by regulatory agencies based on these preliminary data led to uncertainty about the appropriate approach to DTG use among adolescent girls and women of reproductive potential, affecting plans for global DTG roll‐out, and limiting DTG use in adolescent girls and women. This response, along with feedback following the July 2018 WHO interim guidance on DTG, also revealed a critical tension between individual choice and a public health approach to ART guidelines, in which a simplified approach relying on use of the same ART regimen across all adolescents and adults living with HIV is used to allow universal treatment access within the context of healthcare systems with highly constrained capacity and resources 5. Additionally, this situation highlighted difficulties in interpretation and clear messaging of an unexpected safety signal with uncertainty about risk. This was compounded by the lack of comprehensive high‐quality data on pregnancy outcomes from women receiving preconception DTG at sites outside of the Tsepamo surveillance study or countries outside of Botswana, including low rates of reporting to the existing Antiretroviral Pregnancy Registry (APR) 6. This lack of data has resulted in a prolonged period of uncertainty while data on additional exposures are evaluated to refute or confirm the initial safety signal.

Regardless of whether the DTG NTD signal diminishes or is refuted with increased data collection, a better framework for evaluating and introducing new drugs is needed to ensure appropriate data collection to inform the drug safety in adolescent girls and women of reproductive potential and minimize confusion when a safety signal is identified. This challenge is not limited to HIV drugs; many women need treatment for chronic medical conditions such as epilepsy, diabetes and hypertension; should women with such conditions become pregnant, continuation of their medications is often required for both foetal and maternal health, despite the frequent lack of data on safety in pregnancy. This lack of data can result in confusion and difficulty if a safety signal is identified. Additionally, drugs may need to be used despite the presence of known risks (e.g. valproic acid may be needed to treat a epilepsy despite a known association with NTD) 7.

## 2 Discussion

### 2.1 Proposed principles for assessing new safety signals

Critical to the evaluation of safety signals are the quality, interpretability and comparability of additional data, including the settings in which the data are collected (e.g. the prevalence of NTDs varies significantly between countries with and without food folate fortification); the ability to weigh the magnitude and severity of a potential safety risk in relation to the magnitude and degree of all potential drug risks and benefits; the appropriate messaging of drug risks and benefits in pregnant women and those of reproductive potential; and involvement of the affected community in such messaging.

### 2.2 Earlier availability of preclinical reproductive toxicity data

Currently, there is limited evidence to inform treatment decisions for women who are pregnant or who may become pregnant. Data from reproductive toxicology studies in animal models have been used to screen for potential developmental/reproductive hazards of new drugs. However, completion of reproductive toxicity preclinical studies is not required until phase III clinical trials are underway; in the absence of such data, pregnant women are excluded from clinical trials.

However, while negative preclinical reproductive toxicology test results are reassuring, there is no assurance that negative results obtained by testing drugs in animals can definitively predict that a drug will lack teratogenic effects in humans 8. Similarly, it cannot be concluded that agents teratogenic in animals will necessarily produce teratogenic effects in humans. For example, *in utero* EFV exposure at plasma concentrations 1.3‐times that of systemic human therapeutic exposure was associated with central nervous system malformations in cynomolgus monkeys, but many years of prospective human pregnancy outcome data and the recent Tsepamo study findings do not support a teratogenic effect of EFV exposure in human pregnancy 4, 9.

### 2.3 Inclusion of pregnant women in clinical trials

Pregnant women are excluded from most drug trials 10, 11. Pharmacokinetic studies involving pregnant women represent only 1.3% of all phase I trials registered through 2013 12. The general exclusion of pregnant women from clinical trials has been predicated on concerns related to the potential foetal harms of medication used during pregnancy; this fails to consider the risk of withholding optimal therapies from pregnant women because of lack of data. Additionally, general exclusion of pregnant women from clinical trials shifts the risk from the clinical trials setting, where there is intensive safety monitoring, to the clinical care setting, where monitoring is usually minimal. The resultant lack of a solid evidence‐base on drugs in pregnancy puts pregnant women at risk of potentially harmful interventions, suboptimal treatment and/or failed prevention of maternal disease 13. Thus, once initial phase I/II studies in non‐pregnant adults have ruled out substantial safety issues with a new drug and identified appropriate dosing, it is critical that studies of new drugs that will be used by women of reproductive potential undergo phase I pharmacokinetic and safety studies in pregnancy prior to, instead of many years after, drug approval 14.

### 2.4 Improved post‐marketing surveillance and sentinel site surveillance

The evaluation of the potential association of a drug with birth defects (and other uncommon adverse pregnancy outcomes such as stillbirth) necessarily requires adequate post‐drug approval surveillance, because even with early pharmacokinetic studies, too few pregnant women will have drug exposure prior to approval to evaluate an infrequent outcome. To rule out a twofold increase in overall birth defect risk, with a 3% prevalence in the general population, 200 preconception/early first trimester exposures are required; however, for rare defects like NTDs (0.1% and ≤0.06% prevalence in countries without and with food folate fortification respectively), at least 2000 preconception/early first trimester exposures are needed to rule out even a threefold increase in risk (e.g. from 0.1% to 0.3%) 15, 16. Thus, it is only possible to determine the specific risk of birth defects or other rare events by having an appropriate surveillance system in place as new drugs are introduced in populations including women of reproductive potential.

Post‐marketing surveillance to evaluate safety in pregnancy needs to be of high quality and rapidly generated. Pharmacovigilance databases such as the FDA Adverse Event Reporting System (FAERS) and European Medicines Agency EudraVigilance include exposure data collected only after the event has occurred 17, 18. This retrospective enrolment introduces potential bias, with lack of a denominator for the number of patients exposed to the drug of interest. Other shortcomings of these databases include duplicate case reports both within and between databases that may be difficult to identify and limited information about potential confounders 19. For HIV drugs, we must prioritize improvement of reporting of pregnancies and outcomes to voluntary reporting databases such as the APR and European Pregnancy and Pediatric HIV Cohort Collaboration (EPPICC). However, while the APR is an international registry, 74% of reports come from the United States and its territories and the EPPICC study focuses on Europe 6. Short of compulsory reporting, funding of additional sentinel site surveillance (like the Tsepamo study) from low‐ and middle‐income countries, where most HIV‐positive women of reproductive age reside and exposures of concern will occur, is essential. For example, PEPFAR continues to fund birth outcomes surveillance like the Tsepamo study in Uganda and Malawi, which will be important as new antiretroviral drugs are introduced into national programmes 20. A standardised variable list and data collection process would allow for appropriate pooling of data and comparisons across such databases; for example, the WHO has developed tools, data forms and training to assist countries in the development of birth defect surveillance 21. Depending on the drug and extent of anticipated use in pregnancy, there should be a commitment to evaluating a fixed number of pregnancy outcomes with preconception exposure to detect rare events within a specific time after drug approval. Sentinel site surveillance in geographic areas where high use of the drug of concern is anticipated would be important to reach this goal.

### 2.5 Comprehensive analysis of risks and benefits

No drug is completely without potential risk for the mother/foetus; however, untreated or sub‐optimally treated maternal disease may pose a greater risk to the mother and her developing fetus than maternal use of the drug 13. Modelling is a valuable additional modality for providing comparative longer‐term assessment of risk, benefits, and impact of different policies in a variety of settings, using sensitivity analyses when risk remains uncertain. For example, two groups have used modelling to compare use of DTG‐based to EFV‐based ART in women of reproductive potential, evaluating risks and benefits in settings with differing ranges of fertility, contraceptive availability and use, ART efficacy, tolerability, and antiretroviral drug resistance, and differing NTD risks with preconception DTG exposure 22, 23. Both studies enumerated the trade‐offs in terms of risk of NTDs and infant death (which may be higher with DTG) versus risks of maternal disease progression, maternal death, perinatal HIV transmission, and transmission to sexual partners for people living with HIV (which may be higher with EFV due to lower tolerability and less rapid viral replication suppression). By explicitly quantifying these possible trade‐offs, models can help inform discussion of risks and benefits at the population level, but policy decisions must also account for context‐specific and individualized discussions regarding the relative weighing of each of these potential outcomes for women and their children.

With a safety signal there are accompanying regulatory obligations to release a safety report to healthcare providers and the public. However, from a guidelines perspective, in the absence of a clear or impending public health emergency, a comprehensive risk‐benefit evaluation should be performed to put the safety signal in context, including drug availability and need, its effectiveness, toxicity, tolerability, cost and specific characteristics (e.g. antiretroviral drug resistance barrier and profile). This evaluation can both inform and frame the data analysis in terms of impact on individuals and programmes and should be incorporated into any deliberations over recommendations to be included in public health guidelines.

### 2.6 Effective counselling on risks and benefits for women of reproductive potential

In studies evaluating patient counselling on potential drug teratogenic risks, women of reproductive potential have identified several elements of effective counselling. Key elements include information about the effect of a prescribed drug on future consequences for their reproductive health regardless of their current pregnancy intentions; clear and accessible information regarding potential risks as well as potential benefits of the medication, with repeated emphasis on important information; and the communication of information in a private manner with sufficient time to discuss questions with the provider 24. Tools to assist the healthcare provider with short, concise counselling messages would be important for high‐burden settings where time for a patient‐centered approach may be limited.

### 2.7 Critical role of the affected community

A key consideration when a safety signal is identified is the role of the affected community in decision‐making processes. Women living with HIV have strongly expressed the importance of ensuring a woman's right to make her own informed choice among ART regimen options, and that women should not be denied access to a beneficial and preferred antiretroviral drug based on their reproductive potential 25. It is crucially important that community engagement and support are sought before recommendations are made to policymakers, stakeholders and governments. Early and continued community consultation and engagement should be part of a safety signal evaluation and any subsequent guidance and policy recommendations.

### 2.8 Flexibility of guidelines process, allowing for rapid revision

Guidelines are vitally important in interpreting data and transforming it into recommendations or policy. Following the uncertainty engendered by the initial reaction to the DTG safety signal, national guidelines varied considerably in their recommendations on DTG use in women 5, 25. Guidelines committees should not rely on a safety signal alone for rare events but should consider this possible risk within a larger context, including all relevant risks and benefits as well as the input of people requiring the drug therapy under consideration. Specifically, the WHO Guidelines process includes considerations of efficacy, safety, the certainty of evidence, cost, values and preferences of patients and providers, feasibility, equity and human rights 24. When an unexpected safety signal arises, as occurred with DTG in 2018, guidelines processes need to be flexible to enable rapid collection of new information to confirm or refute the signal, to provide considerations for guidance in the case of a prolonged period of uncertainty, provide sufficient time to include a consultative process with the affected community to avoid guidance which may result in controversy or uncertainty, and allow for rapid revision of guidance as soon as important new information becomes available.

### 2.9 Need for clear messaging on risk/benefit for a range of stakeholders

The public health approach to ART has been predicated on simplicity and task‐sharing with health care workers with less advanced training. A potential but not yet confirmed safety signal introduces a layer of complexity that may be difficult to implement. For example, pending further data to confirm or refute the DTG safety signal, the current WHO approach calls for women of reproductive potential to make an informed choice regarding their ART regimen. Health care workers are likely to need additional training and sufficient time with each patient to deliver complicated individual counselling messages in a consistent way. The ability to make an informed choice relies upon having alternative regimens available; however, such alternatives are also required in the event of other toxicities, and need to be accounted for when countries are planning programmes.

In parallel with guidance and policy there is a need for clear messaging. This needs to provide a range of levels of simplicity/complexity depending on the audience 26. Messaging should achieve uniformity across agencies, including messaging for country Ministries of Health to help governments implement strategies and avoid ambiguity in interpretation. Patients and healthcare providers need to have appropriate and clear information and materials 27, 28. They need to be able to assess patient‐specific treatment options, communicate risk and benefits including levels of certainty, and ways to potentially mitigate risk as well as support women‐centered decision‐making. Training in these domains is an essential element of appropriate messaging; increasing treatment literacy is urgently needed and requires investment and funding.

## 3 Conclusions

The response to a rare safety signal in pregnancy needs to be rapid and comprehensive. Such a response requires the preceding existence of appropriately designed surveillance systems with excellent data quality; data analyses that examine risk‐benefit trade‐offs in a variety of contexts; guidance that can transform this risk‐benefit balance into effective and agreed‐upon policy; involvement of the affected community and other key stakeholders; and a communication plan for all levels of knowledge and complexity. The involvement of the community, public health and governmental agencies, guideline committees, Ministries of Health, academics, funders, and stakeholders is essential in order to avoid uncertainty and differing interpretation, and their consequences for providers and patients. Such systems urgently need to be put into place so that any drug used in pregnancy can be evaluated rapidly when introduced and as soon as possible once a serious a safety alert arises. This will require collaborative efforts and provision of resources from diverse stakeholders, including public health and governmental agencies such as WHO, PEPFAR and ministries of health; regulatory agencies; pharmaceutical companies; and the affected community.

## Competing interests

IM, ANP, GKS, JW, AH, DHW, AC, AF, PC, ER, KS, PE and SM report no competing interest. LM is a paid consultant to review dolutegravir safety in pregnancy for the World Health Organization. CT has received funding from ViiV Healthcare via the PENTA Foundation for a pharmacovigilance study on dolutegravir use in pregnancy. AP has received support from ViiV. Healthcare, Gilead Sciences Merck Inc and Janssen for research. SK has received support from ViiV Healthcare, Gilead Sciences Merck Inc and Janssen for research and for the Liverpool Drug Interactions resource.

## Authors’ contributions

AP, JW, ER and PE initiated the idea for and convened the Forum meetings. LM wrote the initial draft of the paper, incorporated comments and developed the final paper draft. All co‐authors participated in the Forum meetings and have made substantial contributions through critical review and extensive comments on drafts of the paper.

## Supporting information


**Appendix S1.** Participants of the International AIDS Society Forum on Dolutegravir Safety.
