# Supplementary material for: Optimizing responses to drug safety signals in pregnancy: the example of dolutegravir and neural tube defects
Source: J Int AIDS Soc. 2019 Jul 12;22(7):e25352. doi: 10.1002/jia2.25352 (PMC6625340; doi:10.1002/jia2.25352)
Supplement: Supplementary file 1 — Appendix S1. Participants of the International AIDS Society Forum on Dolutegravir Safety. [file JIA2-22-e25352-s001.docx]

**Appendix:** Participants of the International AIDS Society Forum on Dolutegravir Safety:

| **Name** | **Organisation** |
| --- | --- |
| Anton Pozniak | Convener Chair: International AIDS Society, President; Chelsea and Westminster Hospital, LSHTM, London, UK |
| Jacque Wambui | Convener Co-Chair: National Empowerment Network of people living with HIV/AIDS in Kenya (NEPHAK) and AfroCAB, Nairobi, Kenya |
| Elliott Raizes | Convener Co-Chair: Centers for Disease Control and Prevention, Atlanta, GA, US |
| Nahida Chaktoura  Andrea Ciaranello | National Institutes of Health, Bethesda, Maryland, United States  Cost-Effectiveness of Preventing AIDS Complications (CEPAC ) Modelling Group, Massachusetts General Hospital, Boston, MA, US |
| Polly Clayden | iBase, London, UK |
| Esther Dixon-Williams | European AIDS Treatment Group |
| Caitlin Dugdale | Cost-Effectiveness of Preventing AIDS Complications (CEPAC ) Modelling Group, Massachusetts General Hospital, Boston, MA |
| Meg Doherty | World Health Organization, Geneva, Switzerland |
| Peter Ehrenkranz | Bill and Melinda Gates Foundation, Seattle, WA, US |
| Ade Fakoya | The Global Fund, Geneva, Switzerland |
| Nathan Ford | World Health Organization, Geneva, Switzerland |
| Andrew Hill | University of Liverpool, Liverpool, UK |
| Saye Khoo | University of Liverpool, Liverpool, UK |
| Maggie Little | Georgetown University, Washington DC, US |
| Imelda Mahaka | Pangaea Zimbabwe AIDS Trust (PZAT), Harare, Zimbabwe |
| Caroline Middlecote | Clinton Health Access Foundation |
| Surbhi Modi | Centers for Disease Control and Prevention, Atlanta, GA, US |
| Lynne Mofenson | Elizabeth Glaser Pediatric AIDS Foundation, Washington DC, US |
| Cynthia Moore | Centers for Disease Control and Prevention, Atlanta, GA, US |
| Wame Mosime | The International Treatment Preparedness Coalition (ITPC) |
| Andrew N Phillips | University College London, London, UK |
| Mimi Raesima | Botswana Ministry of Health, Gaborone, Botswana |
| Virginia Rasi | University College, London, UK |
| George K Siberry | US Agency for International Development (USAID), Arlington, VA, US |
| Kenly Sikwese | African Community Advisory Board (AfroCAB) |
| Claire Thorne | University College London Institute of Child Health, London, UK |
| D Heather Watts | Office of the Global AIDS Coordinator (OGAC), US State Department, Washington DC, US |

*The International AIDS Society Forum on Dolutegravir Safety was designed to create a swift and time-limited process to address how to respond to public health concerns related to use of dolutegravir-containing regimens in women of childbearing potential. The forum was not meant to develop a set of considerations which could be applied when a potential safety signal is identified for a drug in women of childbearing potential. The Forum met in person in September and November 2018. All Forum members contributed to discussion, recommendations and other outputs, but not all Forum members contributed to authorship of this paper.*
